# Informal Face-to-Face Interaction Improves Mood State Reflected in Prefrontal Cortex Activity

**DOI:** 10.3389/fnhum.2016.00194

**Published:** 2016-05-03

**Authors:** Jun-ichiro Watanabe, Hirokazu Atsumori, Masashi Kiguchi

**Affiliations:** Research and Development Group, Hitachi, Ltd.Tokyo, Japan

**Keywords:** face-to-face interaction, mood state, working memory, prefrontal cortex, near-infrared spectroscopy (NIRS)

## Abstract

Recent progress with wearable sensors has enabled researchers to capture face-to-face interactions quantitatively and given great insight into human dynamics. One attractive field for applying such sensors is the workplace, where the relationship between the face-to-face behaviors of employees and the productivity of the organization has been investigated. One interesting result of previous studies showed that informal face-to-face interaction among employees, captured by wearable sensors that the employees wore, significantly affects their performance. However, the mechanism behind this relationship has not yet been adequately explained, though experiences at the job scene might qualitatively support the finding. We hypothesized that informal face-to-face interaction improves mood state, which in turn affects the task performance. To test this hypothesis, we evaluated the change of mood state before and after break time for two groups of participants, one that spent their breaks alone and one that spent them with other participants, by administering questionnaires and taking brain activity measurements. Recent neuroimaging studies have suggested a significant relationship between mood state and brain activity. Here, we show that face-to-face interaction during breaks significantly improved mood state, which was measured by Profiles of Mood States (POMS). We also observed that the verbal working memory (WM) task performance of participants who did not have face-to-face interaction during breaks decreased significantly. In this paper, we discuss how the change of mood state was evidenced in the prefrontal cortex (PFC) activity accompanied by WM tasks measured by near-infrared spectroscopy (NIRS).

## 1. Introduction

In spite of the widespread use of various computer-mediated communication tools, face-to-face communication among team members is still recognized as a key factor for better productivity of the team. Observational or questionnaire-based studies have reported that giving team members opportunities to interact face-to-face contributes to efficient problem solving (Teasley et al., 2000) and the moderation of team empowerment (Kirkman et al., 2004) while enabling employees to perceive each other's personalities in more detail (Hancock and Dunham, 2001) and reduce the feeling of being overloaded (Fussell et al., 1998), and that these in turn can have a positive effect on the team performance. Recent progress with wearable sensors has enabled researchers to study human face-to-face behavior statistically on the basis of rich amount of collected data (Eagle and Pentland, 2006; Olguin et al., 2009). Studies using wearable sensors have revealed a strong relationship between face-to-face interaction among team members and their performance on the basis of quantitative data. In a controlled study, for example, researchers found that the more cohesive the face-to-face communication network, the shorter the time taken to complete a complex task (Wu et al., 2008). The sensors have been used in real-world settings as well to evaluate and improve workplace productivity (Wilson, 2013). One interesting finding from studies at job scenes is that informal face-to-face interaction among employees significantly affects their productivity. For example, in a call center environment, it has been reported that the team's energy and engagement outside of official meetings, defined on the basis of face-to-face behavioral measures obtained by wearable sensors that employees wore, affect their productivity, which is the average handling time (AHT) per call (Pentland, 2012). Another study targeting outbound call centers reported that break time liveliness of the workplace, which is defined by the frequency of employees' bodily movements (caused by face-to-face interaction) measured by accelerometer, had a significant positive effect on their telemarketing sales performance (Watanabe et al., 2012). Another study using wearable sensors targeting school children has also suggested a relationship between break time face-to-face interaction and scholastic performance (Watanabe et al., 2013). All these results point to a strong relationship between informal face-to-face behaviors and productivity on the basis of quantitative data.

However, to our knowledge, the mechanism behind this relationship between informal face-to-face interaction and productivity has not been adequately explained, though it might be well observed and supported by experience at the job scene. We hypothesized that appropriate informal face-to-face interaction, break time chat for example, could improve employees' mood state, thereby affecting their performance. Indeed, many studies have pointed out that psychological states, such as positive or negative mood, affect human performance (Heller and Nitscke, 1998; Gray, 1999; Bolte et al., 2003; Rowe et al., 2007). In mood-cognition interaction studies, working memory (WM; Baddeley, 1992; D'Esposito, 2007) performance has often been evaluated because it has been suggested that WM is affected by some emotional or mood states (Gray, 2001; Shackman et al., 2006). The prefrontal cortex (PFC) has been explored as a target brain region because the PFC is considered to play a crucial role in WM and mood-cognition interaction (Petrides et al., 1993; D'Esposito et al., 1995, 2000; Pessoa, 2008). Previous neuroimaging studies have suggested significant correlation between mood state and PFC activities. A functional magnetic resonance imaging (fMRI) study, for example, showed that unpleasant emotional state, induced by video clips, improved PFC activity during a verbal WM task and impaired activity during a non-verbal WM task, whereas pleasant emotional state exhibited the opposite pattern (Gray et al., 2002). Similarly, a study using near-infrared spectroscopy (NIRS) showed that negative emotion, induced by pictures, increased PFC activity during a verbal n-back WM task (Ozawa et al., 2014). Other studies using NIRS, targeting more natural mood state, have revealed that individuals reporting higher levels of negative mood showed lower levels of PFC activity, especially in the left PFC region, during verbal WM tasks (Aoki et al., 2011; Sato et al., 2011, 2014).

In this study, we aimed to investigate whether informal face-to-face interaction affects mood state and task performance. For subjective evaluation of participants' mood state, we used the questionnaire-based Profiles of Mood States (POMS) method (McNair et al., 1971). For objective evaluation, we used NIRS to measure PFC activity. NIRS has been extensively used in research and for clinical purposes (Maki et al., 1995; Watanabe et al., 1998; Peña et al., 2003; Taga et al., 2003; Suto et al., 2004; Koizumi et al., 2005; Funane et al., 2011; Moriguchi and Hiraki, 2013; Ehlis et al., 2014) because it allows participants to behave more naturally compared to other brain activity measuring techniques such as fMRI, which requires fixation of the body during measurement. For our study, it was crucial to have participants behave naturally in order to evaluate the effect of informal face-to-face interaction on mood state and task performance.

## 2. Materials and methods

### 2.1. Participants

Twenty healthy Japanese volunteers participated in this study (5 female and 15 male, mean age ± SD = 36.3 ± 7.5 years). Written informed consent was obtained from all participants after the purpose, procedures, risks, benefits, and voluntary nature of the experiments had been explained to them. This study was approved by the internal review board of the Central Research Laboratory, Hitachi, Ltd. All procedures were in line with the Declaration of Helsinki in its latest version.

### 2.2. Mood state measurement

The participants' mood state was assessed using a short form of the POMS in its Japanese version (McNair et al., 1971; Yokoyama et al., 1990). Participants rated 30 mood-related adjectives on a 5-point scale ranging from 0 (“not at all”) to 4 (“extremely”) on the basis of subjective estimation of their own mood. This rating enabled us to estimate six mood-related measures: Tension-Anxiety (T-A), Depression-Dejection (D), Anger-Hostility (A-H), Vigor (V), Fatigue (F), and Confusion (C). The total mood disturbance (TMD) was then defined as the sum of five negative scores (T-A, D, A-H, F, and C) minus the positive score (V). The higher the TMD score, the more negative the mood of the participant.

### 2.3. PFC activity measurement

**Figure 1:**
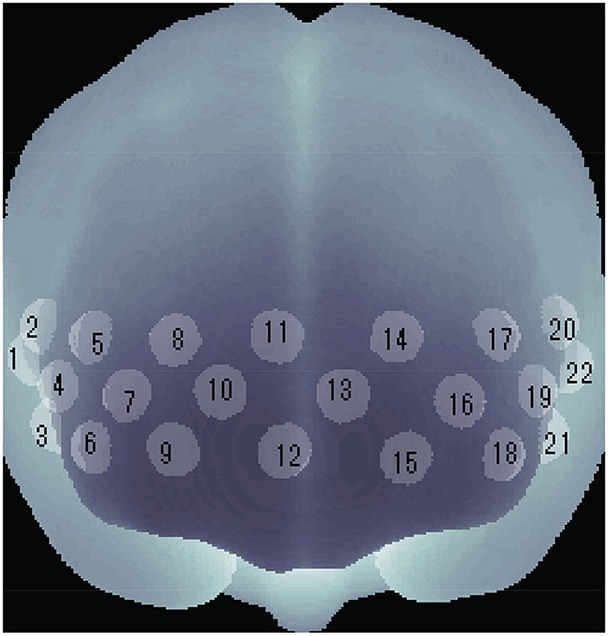
**Channel positions of wearable NIRS device**. Each position is defined as the center point of each light source and detector. Changes in Hb concentration were estimated at each channel.

We used a multi-channel NIRS (optical topography) system (Atsumori et al., 2009, 2010) for measuring the cerebral blood volume change of the participants. The NIRS system generally measures the change in the product of hemoglobin (Hb) concentration and effective optical path length in human brain tissue. The unit of Hb change is molar concentration (mM = mmol/l) multiplied by optical path length (mm). The NIRS device emits infrared light at two wavelengths, 754 and 830 nm, to the scalp, and detects reflected light once every 200 ms. The changes of oxygenated (oxy-Hb) and deoxygenated (deoxy-Hb) hemoglobin are estimated by using absorption coefficient spectra for the two wavelengths according to the modified Beer-Lambert law (Delpy et al., 1988; Maki et al., 1995). The NIRS device covers the entire forehead. The 22 channel positions of the NIRS device are shown in Figure 1.

### 2.4. Working memory tasks

**Figure 2:**
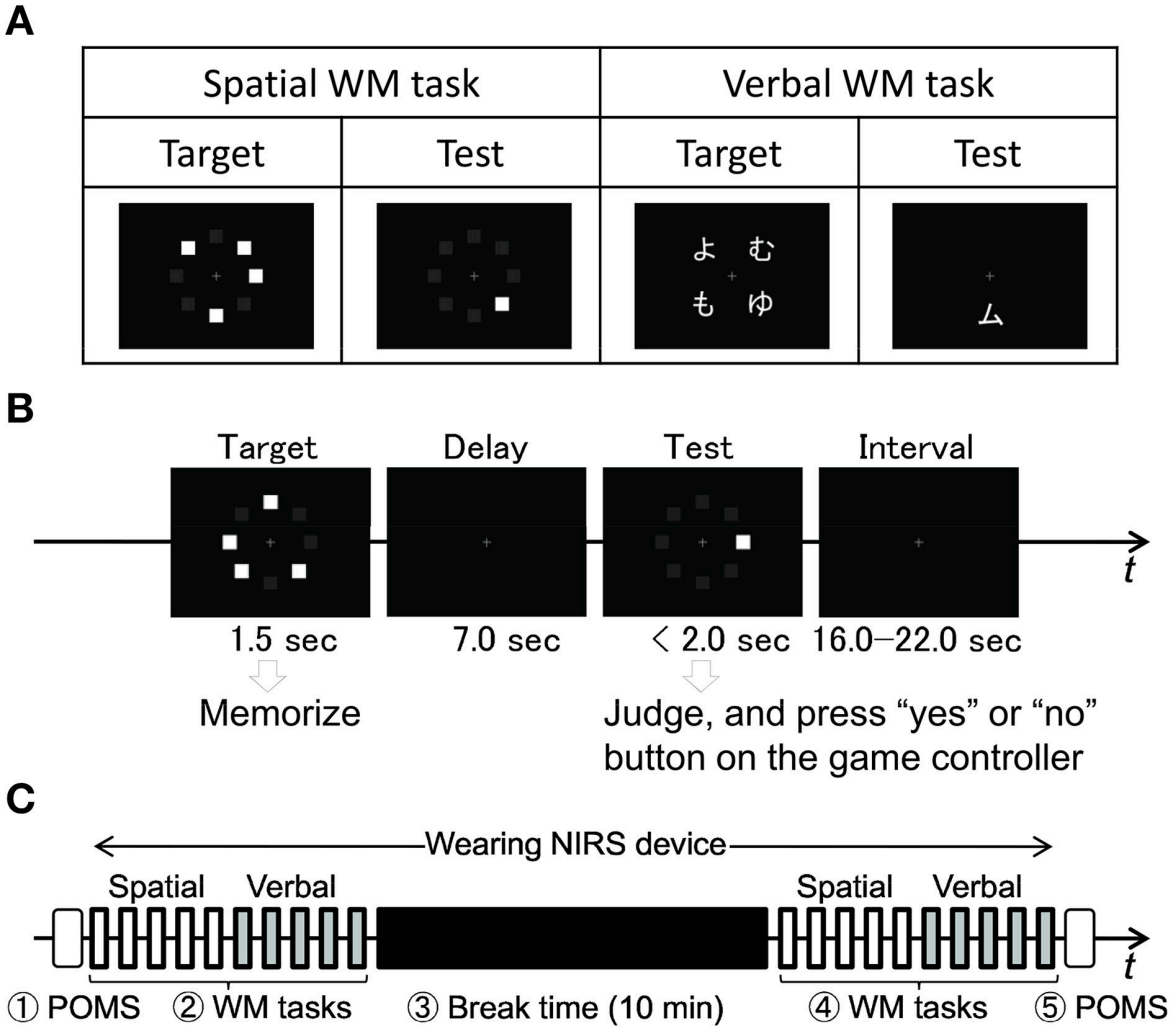
**WM tasks and experimental procedure**. **(A)** Examples of Target and Test stimuli for spatial and verbal WM task. **(B)** A task is composed of presenting a Target followed by a delay and presenting a Test followed by an interval before the next task trial. Participant responded by pressing button on game controller judging whether Test stimuli corresponded to one of the Target stimuli. **(C)** Time sequence of POMS and WM task trials including 10 min break. Participants remained sitting at a desk in a room facing a laptop for task presentation throughout the experiment, which took about 30 min to complete. All participants wore NIRS device continuously during WM tasks and break times.

We used verbal and spatial delayed match-to-sample WM tasks (Smith et al., 1996) to measure PFC activities (Figure 2A). These were basically the same as the ones used in previous NIRS studies (Aoki et al., 2011; Sato et al., 2011, 2014). Each task trial started with a 1.5 s presentation of the target stimuli (Target) on a laptop screen (Figure 2B). After presenting a delay (black screen with white fixation cross) for the following 7.0 s, a test stimulus for retrieval (Test) was then presented for 2.0 s or until the participant responded. The participant responded by pressing a button on a handheld game controller (ELECOM Co., Ltd., JC-U2312F) connected to the laptop. For the verbal WM task, a set of four Japanese *hiragana* characters was presented as the Target and a Japanese *katakana* character was presented as the Test. Participants were asked to judge whether the Test character corresponded to one of the four Target characters. They used right index and middle fingers to press “yes” and “no” buttons, respectively, for the judgment. Because the characters presented as Target and Test were in different Japanese morphograms, participants made their decisions on the basis of the phonetic information conveyed by the characters, not on the basis of their form. Auditory cues (1000 and 800 Hz pure tones of 100 ms duration) were presented at the Target and Test onsets, respectively. For the spatial WM task, Target was given by the locations of four squares irregularly arranged at eight peripheral locations. Test was given by a square presentation at one of the eight locations. Participants were asked to judge whether Test square location corresponded to one of the Target locations.

### 2.5. Experimental procedure

We had each participant engaged in the experiment one day between 10 and 11 am. The experiment was conducted in a small room (approximately 5 × 5 m) with window blinds blocking sunlight and fluorescent lights providing lighting source to maintain the same brightness to each participant. The room temperature was also kept the same by the air conditioner. Participants were asked to sit at a desk facing a laptop for the WM task presentation for the duration of the experiment, which took about 30 min to complete. A supervisor remained in the same room for dealing with unexpected system troubles or taking care of any health issues the participants might experience, though NIRS system has a high level of safety (Ito et al., 2000; Kiguchi et al., 2007). The participants were divided into two groups: *G*_*alone*_ and *G*_*f*2*f*_. *G*_*alone*_ was with 9 participants, all male, who went through the experiment alone with a supervisor. *G*_*f*2*f*_ was with 11 participants who were paired to go through it with a supervisor in the same room. The paired participants included 2 female-female and 1 female-male pairs.

The experiment consisted of five sequential procedures (Figure 2C). First, the participant filled in an initial POMS questionnaire to measure his or her mood state at the time. This took about 2 min. Second, the participant put on a NIRS device and performed the WM tasks. These tasks were composed of five spatial WM tasks and five verbal WM tasks presented sequentially with intervals from 16.0 to 22.0 s. It took about 5 min to complete all 10 WM tasks. Third, participants took breaks for 10 min while continuing to wear the NIRS device. During the break, they were asked to remain sitting but otherwise to behave as they liked. Four magazines were placed on the desk free to be read. For each participant in *G*_*alone*_, the supervisor left the room during breaks to make the participant actually alone. Some participants in *G*_*alone*_ spent their break time reading the magazines, while others did nothing special. The participants in *G*_*f*2*f*_ were urged to chat with the other people. The supervisor took the initiative in starting the face-to-face chats. Fourth, after the break, participants performed the 10 WM tasks again, which this time had different content. Finally, participants were asked to take off the NIRS device and fill out the POMS questionnaire again to measure their current mood state.

### 2.6. NIRS data analysis

For the NIRS data analysis, we used MATLAB (The MathWorks, Inc., U.S.A) and plug-in-based software developed in-house. We defined a block for each signal as the period from 1.0 s before presenting Target (which lasts 1.5 s, followed by 7.0 s delay for memorization) to 16.0 s after starting presenting Test, for 25.5 s in total. We conducted five blocks of spatial WM tasks and five blocks of verbal WM tasks both before and after the break (see Figure 2C). To remove components originating from slow fluctuations of blood flow, a 1.0 Hz low pass filter was applied to the signals. Then, for the oxy-Hb signal in each block, a first-degree baseline-fit was estimated to set the first 1.0 s to the level of zero. Moreover, all blocks that included oxy-Hb signals exceeding the range from −0.20 to 0.25 mM·mm were removed, as such blocks might have been affected by movement artifacts. Finally, we defined the “PFC activity period” as the period from 5.0 s after presenting Target to just before starting presenting Test (3.5 s) to investigate the signal change avoiding effects related to the motor response (Aoki et al., 2011). Changes in oxy-Hb concentration during the PFC activity period were then estimated at each channel.

## 3. Results

### 3.1. Mood state

**Figure 3:**
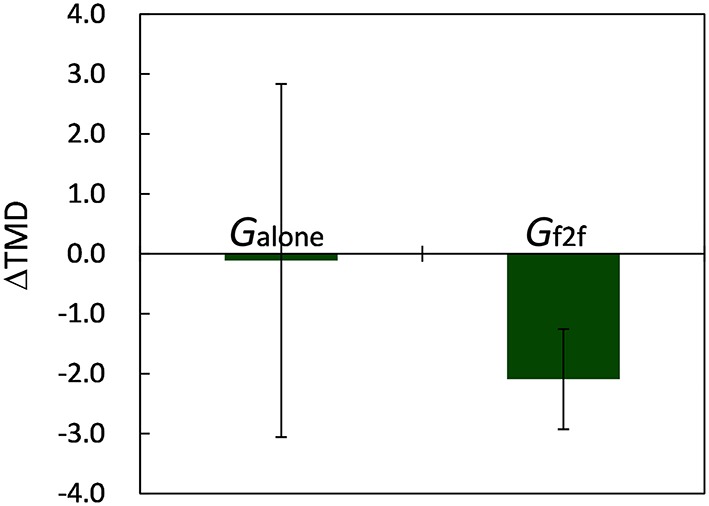
**Changes of TMD**. Though the mean difference between the two groups was not significant (*p* > 0.48), participants in *G*_*f*2*f*_ improved their mood state significantly (*p* < 0.04), whereas those in *G*_*alone*_ did not. Error bars represent SEM.

The average scores of six mood-related measures and TMD for *G*_*alone*_ and *G*_*f*2*f*_ calculated before and after the 10 min break are summarized in Table 1 (the scores for each participant are provided in Data Sheet S1). Hereafter, “before” and “after” refer to events before and after the break, and measures are denoted with these suffixes. We observed no significant difference between the mood states of *G*_*alone*_ and *G*_*f*2*f*_ before starting the experiment [*t*_(18)_ = 0.44, *p* > 0.65 for *TMD*_*before*_, Student's *t*-test]. After the break, we observed that the mood states of participants in *G*_*alone*_ did not change, whereas those in *G*_*f*2*f*_ significantly improved: *t*_(10)_ = 2.50 (*p* < 0.04) for TMD, 3.19 (*p* < 0.01) for A–H, and 3.31 (*p* < 0.01) for *F* (paired *t*-test). The V scores were worsened for both groups [*t*_(8)_ = 2.51 for *G*_*alone*_ and *t*_(10)_ = 2.61 for *G*_*f*2*f*_, *p* < 0.04 for both]. This could be due to tiredness following the 30 min of experiment. The changes of TMD, defined as ΔTMD≡TMD_after_−*TMD*_*before*_, were −0.11±8.84 for *G*_*alone*_ and −2.09±2.77 for *G*_*f*2*f*_ (mean ± SD, Figure 3). Though the mean difference was not significant (*p* > 0.48), this indicates that most of the participants in *G*_*f*2*f*_ improved their mood state, whereas some of the participants in *G*_*alone*_ worsened.

**Table 1:** **Changes of POMS scores**.

	***G***_*****alone*****_		***G***_*****f***2***f*****_	
	**Before**	**After**	**Before**	**After**
T-A	6.7±5.7	6.6±5.0	4.4±3.0	3.8±2.4
D	3.6±3.6	2.4±3.7	2.7±3.2	2.1±3.1
A-H	0.6±1.1	0.2±0.4	**3.0**±**1.7**	**2.0**±1.7^*^
F	4.4±3.6	4.3±3.8	**5.4**±**3.9**	**4.1**±3.7^*^
C	7.1±2.7	6.7±2.9	5.2±2.4	4.8±2.4
V	**7.0**±**5.7**	**4.8**±7.0^**^	**7.6**±**3.8**	**5.9**±4.6^**^
TMD	15.6±11.5	15.5±12.7	**13.2**±**12.1**	**11.1**±11.9^**^

All scores except V represent degree of negative mood state, so the larger value, the more negative the mood. Values are mean ± SD. ^*^ and ^**^ indicate significant differences from “Before.”

* *p < 0.01*,

** *p < 0.05*.

*Bold values emphasize significant differences*.

### 3.2. PFC activity during WM tasks

We focused on the oxy-Hb signals in our analysis, as previous NIRS studies on the relationship between mood states and PFC activities found their main results in this signal (Aoki et al., 2011; Sato et al., 2011, 2014). We used the mean change of oxy-Hb signals during the PFC activity period over the five sequential blocks for spatial and verbal WM tasks, denoted here as *Hb*^*spa*^ and *Hb*^*verb*^, respectively, as the measure estimating the PFC activity. The values for each participant are provided in Data Sheet S2. At several channels for some participant, we failed to detect enough light, possibly because the channel positions corresponded to haired regions. We denote the number of data used in the analyses as *n*_*alone*_ and *n*_*f*2*f*_ for the two groups, respectively.

**Figure 4:**
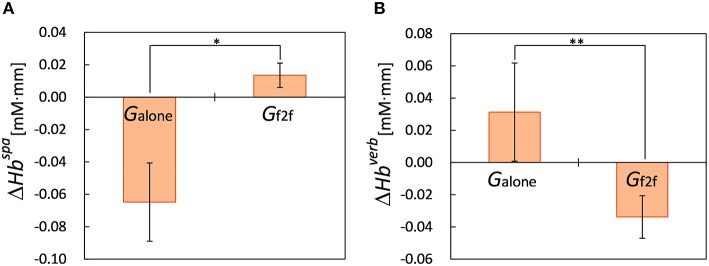
**Changes of PFC activity during WM tasks**. **(A)** PFC activity of *G*_*alone*_ during spatial WM task decreased significantly at ch 4 (*p* < 0.04) after the break whereas that of *G*_*f*2*f*_ did not. We observed significant difference between PFC activity changes, Δ*Hb*^*spa*^, of the two groups at this channel (^*^p < 0.01). **(B)** PFC activity of *G*_*f*2*f*_ during verbal WM task decreased significantly at ch 13 (*p* < 0.04) after the break whereas that of *G*_*alone*_ did not. We observed significant difference between PFC activity changes, Δ*Hb*^*verb*^, of the two groups at this channel (^**^p < 0.05). Error bars represent SEM.

We observed that for participants in *G*_*alone*_, their PFC activity during spatial WM task significantly decreased after the break at ch 4 (*p* < 0.04, *n*_*alone*_ = 8), which is in right PFC region. Moreover, we found significant difference between the changes of PFC activity during spatial WM tasks, $\Delta Hb^{spa}\equiv Hb_{\text{after}}^{spa}-Hb_{\text{before}}^{spa}$, of the two groups at this channel (Δ*Hb*^*spa*^ = −0.06±0.07 mM·mm for *G*_*alone*_, 0.01±0.02 mM·mm for *G*_*f*2*f*_, *p* < 0.01, *n*_*f*2*f*_ = 9, mean ± SD, Figure 4A).

For the PFC activity during verbal WM tasks, in contrast, we observed opposite pattern. We observed that for participants in *G*_*f*2*f*_, their PFC activity during verbal WM task significantly decreased after the break at ch 13 (*p* < 0.04, *n*_*f*2*f*_ = 8), which is in middle left PFC region. We found significant difference between the changes of PFC activity during verbal WM tasks, $\Delta Hb^{verb}\equiv Hb_{\text{after}}^{verb}-Hb_{\text{before}}^{verb}$, of the two groups at this channel (Δ*Hb*^*verb*^ = 0.03±0.07 mM·mm for *G*_*alone*_, *n*_*alone*_ = 5, −0.03±0.04 mM·mm for *G*_*f*2*f*_, *p* < 0.05, mean ± SD, Figure 4B). We also observed that after the break, PFC activities of *G*_*alone*_ during verbal WM task were significantly greater than those of *G*_*f*2*f*_ at ch 16 (*p* < 0.05, *n*_*alone*_ = 5, *n*_*f*2*f*_ = 8) and at ch 18 (*p* < 0.01, *n*_*alone*_ = 8, *n*_*f*2*f*_ = 9), which are in left PFC regions. There were no significant differences between the PFC activities of the two groups at these channels before the break.

### 3.3. Task accuracy

**Figure 5:**
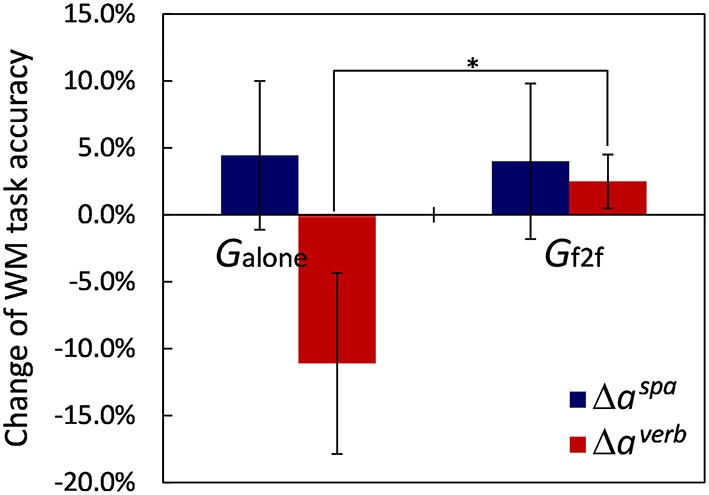
**Changes of WM tasks accuracies**. Verbal WM task accuracy of *G*_*alone*_ decreased after the break and showed remarkable difference for the change in *G*_*f*2*f*_ (^*^p = 0.059). Error bars represent SEM.

We used the mean accuracy of the five sequential WM tasks of spatial and verbal contents, here denoted as *a*^*spa*^ and *a*^*verb*^, respectively, as measures representing task performance. The values for each participant are provided in Data Sheet S3. For this analysis, we removed one participant belonging to *G*_*f*2*f*_ because of a failure to detect his button press event. Figure 5 shows the average changes of the task performance, $\Delta a^{spa}\equiv a_{\text{after}}^{spa}-a_{\text{before}}^{spa}$ and $\Delta a^{verb}\equiv a_{\text{after}}^{verb}-a_{\text{before}}^{verb}$, for the two groups. Before taking breaks, we found no significant differences between the task performances of the two groups: *t*_(17)_ = 0.46, *p* > 0.65 for $a_{\text{before}}^{spa}$, and *t*_(17)_ = 1.41, *p* > 0.17 for $a_{\text{before}}^{verb}$. After the break, for the spatial WM task performance, we observed that the mean accuracy slightly improved for both groups (Δ*a*^*spa*^ = 4.4±16.6% for *G*_*alone*_ and 4.0±18.3% for *G*_*f*2*f*_, mean ± SD), and that the difference between the groups was not significant (*p* > 0.95). This slight increase could be due to the practice the participants got from the first trial before the break. For verbal WM task performance improvement, in contrast, we observed a remarkable difference between the two groups [*t*_(17)_ = −2.02, *p* = 0.059]: Δ*a*^*verb*^ = 2.5±6.3% for *G*_*f*2*f*_ (with improved mood states), whereas it was −11.1±20.2% for *G*_*alone*_ (with no mood state change). These suggest a relationship between participant mood state and verbal WM task performance.

In fact, we observed significant correlations between individual mood state and verbal WM task accuracy. Specifically, we observed that *TMD*_*after*_ had a significant negative correlation with $a_{\text{after}}^{verb}$ and Δ*a*^*verb*^ (Pearson's correlation coefficient: *r* = −0.51, *p* < 0.03, and *r* = −0.54, *p* < 0.02, respectively). In addition, we observed that the change of F score, Δ*F*≡*F*_*after*_−*F*_*before*_, also correlated significantly with Δ*a*^*verb*^ (*r* = −0.52, *p* < 0.03). These indicate that participants whose mood states were improved after the break improved their performance and had higher verbal WM task accuracy. We observed no such correlations between mood state and spatial WM task accuracy.

## 4. Discussion

The importance of face-to-face interaction among team members for better team productivity has been well recognized by experience and observed in previous psychological and social studies (Guzzo and Shea, 1992; Stewart, 2006). Recent studies using wearable sensors have also provided evidence of the relationship between informal face-to-face interaction and productivity on the basis of quantitative data (Pentland, 2012; Watanabe et al., 2012, 2014). However, the mechanism behind this relationship has not been well explained.

On the basis of subjective POMS results, we can consider that after the break, participants in *G*_*f*2*f*_ had improved (somewhat pleasant) mood state whereas those in *G*_*alone*_ kept being in comparatively unpleasant mood state. We observed that after the break, participants belonged to *G*_*f*2*f*_ significantly decreased PFC activity during verbal WM tasks while keeping the high task accuracy, whereas those in *G*_*alone*_ did not. This suggests that after the break, comparatively less load was applied on verbal WM for participants in *G*_*f*2*f*_, while for participants in *G*_*alone*_, the verbal WM tasks were difficult enough to urge PFC activity to be still active. The current NIRS data also showed that PFC activity during the spatial WM task decreased significantly after the break for *G*_*alone*_ and did not for *G*_*f*2*f*_. These results are in line with an fMRI study suggesting that the emotional state influences task-related brain activity (Gray et al., 2002). In their study, they suggested that the greatest neural activity was for the verbal WM task with unpleasant emotional state and for the non-verbal task with pleasant emotional state, and the lowest activity was for verbal-pleasant and non-verbal-unpleasant conditions. We also observed that after the break, PFC activities of *G*_*alone*_ during verbal WM task were significantly greater than those of *G*_*f*2*f*_ at left PFC regions. This result is in line with a NIRS study suggesting that negative emotion modulates PFC activity during a WM task (Ozawa et al., 2014). They reported that the oxy-Hb changes during a verbal n-back task were significantly greater following negative rather than neutral stimulation, especially in the left inferior frontal gyrus. The results of our NIRS measurement thus provide objective evidence that participants in *G*_*f*2*f*_ did improve their mood states while those in *G*_*alone*_ did not, suggesting the influence of having or not having informal face-to-face interaction on mood state change and task performance.

However, previous NIRS studies have suggested positive correlation between PFC activity during verbal WM task with mood state especially at left PFC regions (Aoki et al., 2011; Sato et al., 2011, 2014), which is inconsistent with the fMRI and NIRS studies (Gray et al., 2002; Ozawa et al., 2014) we have set as premises. To our knowledge, these contradictory scenarios have not yet been well integrated and are considered to originate in the difference in mood state generation methods, e.g., mood-induction methods or natural conditions. Our current findings suggest that having or not having informal face-to-face interactions might have more impact on mood state (making it positive or negative) than we had expected as targeting a “natural” mood condition.

For spatial WM task performance, we observed neither task accuracy increase after the break nor any correlations with mood state change. This might relate to the fact that previous NIRS studies targeting natural mood condition have failed to observe significant correlation between mood state and PFC activity for the spatial WM task (Aoki et al., 2011; Sato et al., 2011, 2014). Other studies using mood-induction method have pointed out, however, the relationship between unpleasant emotional state and non-verbal task performance (Gray, 2001; Gray et al., 2002). These suggest that spatial WM function might be influenced by relatively intensive emotional state whereas verbal WM is modulated even in the range of natural mood condition, and that the mood state change caused by the break time face-to-face interaction was not strong enough to affect spatial WM task performance.

Our results in this study provide a reasonable explanation for what we observe in the real-world setting. In the call center studies using wearable sensors, for example, it has been revealed that activeness of informal face-to-face interaction among employees plays a key role in their work performance (Pentland, 2012; Watanabe et al., 2012). As we have seen, participants in *G*_*alone*_ failed to improve their mood state, and their verbal task performance significantly decreased. Our results indicate that the mood state of telemarketers, whose work is obviously made up of verbal tasks, is influenced by having or not-having face-to-face interaction with colleagues during breaks, and could relate to their productivity.

Our study has some limitations regarding the experimental settings. We used a small room (approximately 5 × 5 m) throughout the experiment, including the 10 min break. Breaks spent in a larger room for a longer amount of time could potentially affect the degree of mood state change. Moreover, we had participants continue sitting during the break without having any beverages or snacks. Giving them more freedom to behave naturally could also affect the result. For the participants in *G*_*f*2*f*_, we formed teams with three people. The different details of team composition, such as number of members, gender, and age ratios, and/or the difference of personalities, should also be considered. To address these limitations, experiments in the real world must be carried out where the tasks, people, and environment are more diverse.

In our experiment for *G*_*f*2*f*_, the participants did neither talk with each other nor have any interactions during performing the task. However, we realized that not only face-to-face interaction during breaks but also presence of others during the task might affect the mood state and task performance. Actually, previous social facilitation studies pointed out that the presence of others or audience does affect performance, emotion, and/or various human behaviors (Cottrell et al., 1968; Buck et al., 1992; Herman et al., 2003). To evaluate the effects of informal face-to-face interaction on mood state and task performance more explicitly, more careful experimental design, in which participants perform the task alone and spend breaks in a team, is needed.

Finally, the main contribution of this work is our demonstration that informal face-to-face interaction strongly relates to mood state, and that mood state change can be estimated not only by subjective self-reported questionnaires but also by objective brain activity measurement. We believe that our work will lead to further research and a better understanding of the relationship among complex human face-to-face behavior, mood, and cognition.

## Author contributions

Conceived and designed the experiments: JW, HA, MK. Performed the experiments: JW. Analyzed the data: JW, HA. Contributed reagents/materials/analysis tools: HA, MK. Wrote the paper: JW.

## Funding

Financial support for this study was provided by Hitachi, Ltd. The authors are current employees of Hitachi, Ltd. The funder played a role in the study design, data collection and analysis, decision to publish and preparation of the manuscript by providing the budget for this study, data collection system (devices and software), and was involved in discussion about this study.

### Conflict of interest statement

The authors declare that the research was conducted in the absence of any commercial or financial relationships that could be construed as a potential conflict of interest.

## Supplementary material

Data Sheet S1 **The scores of six mood-related measures and TMD for each participant**.

Data Sheet S2 **PFC activity estimated as the mean change of oxy-Hb signals during the PFC activity period over the five sequential blocks for spatial and verbal WM tasks**.

Data Sheet S3 **The mean accuracy of the five sequential WM tasks of verbal and spatial contents**.
