# Supplementary material for: Informal Face-to-Face Interaction Improves Mood State Reflected in Prefrontal Cortex Activity
Source: Front Hum Neurosci. 2016 May 3;10:194. doi: 10.3389/fnhum.2016.00194 (PMC4853847; doi:10.3389/fnhum.2016.00194)
Supplement: Data Sheet S1 — The scores of six mood-related measures and TMD for each participant. [file DataSheet1.PDF]

Data Sheet S1

Mood state

POMS scores

Participants		Before							After						
Group	#	T−A	D	A−H	F	C	V	TMD	T−A	D	A−H	F	C	V	TMD
G_{alone}	p1	1	2	1	0	5	6	3	3	0	0	0	5	0	8
	p2	9	4	0	9	9	6	25	7	5	0	8	9	2	27
	p3	6	3	0	4	6	2	17	0	0	0	0	5	0	5
	p4	15	10	0	3	12	8	32	15	11	0	5	12	4	39
	p5	15	9	2	9	10	15	30	12	2	0	8	10	13	19
	p6	8	1	0	9	7	7	18	10	0	0	9	7	5	21
	p7	7	4	3	4	7	17	8	7	4	1	2	4	20	−2
	p8	0	0	0	1	4	1	4	1	0	0	0	5	0	6
	p9	0	0	0	1	4	1	4	5	0	1	7	4	0	17
G_{f2f}	p10	6	3	5	5	4	10	13	5	3	3	3	3	9	8
	p11	3	0	3	0	4	5	5	4	0	1	1	4	6	4
	p12	4	3	2	6	6	13	8	4	3	2	4	3	14	2
	p13	3	0	1	2	4	4	6	1	0	2	0	4	1	6
	p14	10	7	4	12	8	9	32	6	7	4	11	8	8	28
	p15	2	2	2	6	4	7	9	3	1	0	4	5	0	13
	p16	4	4	5	5	3	14	7	3	1	4	6	4	12	6
	p17	10	10	6	13	11	8	42	9	9	5	11	11	5	40
	p18	1	0	1	4	5	1	10	0	0	0	3	4	0	7
	p19	2	1	3	2	3	6	5	3	0	2	1	3	5	4
	p20	4	0	1	5	6	7	9	4	0	0	2	4	5	5
