# Supplementary material for: Informal Face-to-Face Interaction Improves Mood State Reflected in Prefrontal Cortex Activity
Source: Front Hum Neurosci. 2016 May 3;10:194. doi: 10.3389/fnhum.2016.00194 (PMC4853847; doi:10.3389/fnhum.2016.00194)
Supplement: Data Sheet S2 — PFC activity estimated as the mean change of oxy-Hb signals during the PFC activity period over the five sequential blocks for spatial and verbal WM tasks. [file DataSheet2.PDF]

Data Sheet S2

PFC activity

Spatial Before

Participants		Channels																					
Group	#	ch1	ch2	ch3	ch4	ch5	ch6	ch7	ch8	ch9	ch10	ch11	ch12	ch13	ch14	ch15	ch16	ch17	ch18	ch19	ch20	ch21	ch22
	p1	−0.02104	0.070162	0.018942	0.005281	0.007687	0.013138	0.044036	−0.00929	−0.02099	−0.05805	0.020642	−0.08074	0.10132	0.12252	−0.02971	−0.01869	0.1113	−0.02364	−0.035429	0	0	0
	p2	0.02198	0.02826	−0.0054552	0.051814	0.000583	0.000928			0.025293			0.023374	0.020197	−0.00415	0.017936	0.029038	0.010881	0.035704	−0.012975	0	0	0
	p3			−0.013697	0.065137	0.03002	−0.03996	0.076947	−0.08484	0.072929	−0.09598		−0.00418			0.028109			0.12009		0	8.81E−12	0
	p4				0.093148	0.081875	0.062915			0.15151	0.067267		0.1668	0.17152		−0.02665			0.14749			0	0
G_{alone}	p5	0.013243		0.05736	0.023188	0.075601	−0.03185			0.00953			−0.01289	−0.02388		0.008456		0.031245		0.043208		0.019362	
	p6	−0.00939	−0.08986	−0.035438	0.006096	0.023717	0.005597	−0.0027953	0.000329	−0.00975	0.026873	0.088093	−0.04841	0.10174	0.059797	−0.02194	−0.0298	0.032627	0.008397	0.0045321	0	0	0
	p7			−0.011816	0.078371		0.072768			0.12943	−0.04059		0.093764			0.19881							0.048332
	p8	0.10793	0.063304	0.040188	0.022445	−0.00474	0.010134	0.063572	0.018603	0.06519	0.039113	0.022208	0.069144	−0.0803	−0.04272	0.024536	0.046748	0.12706		−1.17E−05			−0.051037
	p9	0		−0.17805	0.11969	0.17779	0.067149	−0.071963	0.15131	0.114	0.10725	0.17566	0.10615	0.12487	0.11591	0.078526	0.07654		0.031757			0	0
	p10			0.077522			0.11699	−0.012913	−0.01467	−0.03017	0.043269	0.00165	0.012869	−0.01749	0.041541	0.11536	0.075369	0.003417		0.069095	0		0
	p11	−0.0031			−0.02062		0.1061	0		0		0.04939			0.044877		0.14153		0.066018				
	p12	0.095071	0.10602	0.077388	0.059382	0.034532	0.080039	0.10398	−0.00604	0.041039		0.064669	0.052075	0.091089	0.13135	0.11227	0.031845	0.011239	0.042602	0.12244	0	0	0
	p13	−0.03789	0.064736	−0.042324	0.015399	0.066783	−0.01813	−0.007248		−0.00123	0.04347		−0.0129	0.047257		−0.01538				0.10671		−0.027478	−0.000625
	p14			−0.0033314	0.003431	−0.02228	−0.00165		0.04179	−0.02757	0.052408	−0.06609	−0.00415	0.037714	−0.12458	0.097295	−0.03304	0.022002	0.071275	0.024375	0	0	0
G_{f2f}	p15		0.008434	−0.0008435	−0.00579	−0.00627	−0.00252		−0.022	−0.01269	−0.01045	−0.04054	−0.0093	−0.05592	−0.0117	−0.03353							
	p16	0		−7.96E−07			−0.03823	−0.00073	0.032256	−0.0269	0.026541	−0.05261	−0.00384	0.043247	0.031486	0.008772	0.1123	0.041773	−0.00891		−3.06E−14	0	0
	p17	−0.09019	0.074124	−0.0046656	0.006904	0.064514	0.19067		−0.0083	0.036412	0.030418		0.031044			0.073052	0.043532	0.086299	−0.07083				
	p18			−0.030215	0.052498	0.079895	0.03095		0.11025	0.022987	0.042265	0.003655	0.04933	−0.05717	0.086832	0.17469	−0.01626	−0.02891	0.015733	−0.030094			
	p19	0	0	−0.03548	0.023316	−0.02203	0.039873	−0.020333	0.023273	0.056957	0.037907	0.087887	0.031002	0.077731	0.087893	0.031921	0.0367	−0.18449	−0.01845		0	0	0
	p20	0		0	−0.02086	−0.02864	−0.00941	−0.0453	−0.00052	−0.06025			−0.00862	0.048213	0.1044	−0.00588	0.043505			−0.000122	0	0	0

Spatial After

Participants		Channels																					
Group	#	ch1	ch2	ch3	ch4	ch5	ch6	ch7	ch8	ch9	ch10	ch11	ch12	ch13	ch14	ch15	ch16	ch17	ch18	ch19	ch20	ch21	ch22
	p1	−0.00403	−0.05332	−0.018107	0.001041	0.040382	0.017985	0.025455	−0.01438	−0.00783		0.011505	−0.05435		0.054749	−0.02663	0.12516	−0.00542	0.005747	0.019194	0	0	0
	p2	0.037301	−0.01576	−0.081098	−0.10033	0.015527	−0.05383						−0.01313	−0.00286	−0.00459	0.057537	0.10006	0.11764	0.069994	0.034083	0	0	0
	p3	0.071773		0.0075216		−0.01288	0.006459	0.020769	−0.01448	0.063072			0.025498			−0.02543			0.055573		0	−2.49E−05	0
	p4				−0.00815	−0.03214	0.040394	−0.002817		0.12233			0.084376	0.040925		0.10582						0	0
G_{alone}	p5	−0.08168		0.079962	0.052624	0.040302		1.53E−05		0.060975			−0.04132			−0.00265		0.035503		−0.0795		0.14244	
	p6	−0.01673	0.033941	0.0010149	−0.06174	−0.06361	0.021758	−0.091772	−0.01845	−0.04301	−0.01541	−0.02842	−0.02966	−0.02084	0.002371	0.019726	−0.024	0.020242	0.016449	0.0098126	0	0	0
	p7				0.021628		0.1062				−0.03988		0.031982			−0.04628			−0.01738			0.081772	−0.037532
	p8	−0.03121	−0.03414	−0.0034463	0.012081	0.045061	0.025684	0.11232	0.069013	0.041437	0.055175	0.0359	0.052829	0.013261	−0.02197	0.018208	−0.03989	0.033228	−0.09668	−2.25E−16		7.89E−163	2.09E−165
	p9	0		−0.0001474	−0.03524	0.066398	−0.02116	0.010935	0.016108	0.059016	0.027491	0.08852	−0.00058	0.038706	0.015706	−0.01435	−0.0273	−0.06506	0.056483		2.23E−294	0	0
	p10			0.015312			−0.00381	−0.031282	0.015965	0.051402	−0.00286	0.069826	0.12089	−0.01052	−0.00577	0.043877	0.036194	0.038011	−0.0057		0		0
	p11			0.052912	0.027911	0.066264	0.020695	0		0	0.034434				0.18129		0.13319						
	p12	0.027537	0.097362	0.063468	0.06101	0.013906	0.079925	0.05567	0.070963	0.12158	0.060542	0.11648	0.14243	0.063837	0.063597	0.14167	0.041434	0.016497	0.035396	0.035995	0	0	0
	p13	−0.06228	−0.00857	−0.0067477	0.039215	−0.00733	0.029293		−0.0193	−0.02258	−0.014		−0.01526	0.010928		−0.00026			−0.05117	−0.048084		−0.0186	0.042848
	p14			0.013882	0.035788	−0.03113	0.13414		−0.00977	−0.08276	−0.09908	−0.04406	−0.09243		0.080093	−0.01357	0.003878	0.038774	−0.0071	0.015668	0	0	0
G_{f2f}	p15		0.073219	0.12088	0.027394	−0.02697	−0.01046	−1.43E−109	0.015536	−0.03864	−0.05027	−0.06694	−0.05663	−0.02603	0.11231	−0.06572							
	p16	0		−8.05E−295			0.012541	0.087694	−0.00416	0.11976	0.038791	−0.06273	0.01304	0.031292	0.029763	0.050562	0.1326	0.046197	0.023857	0.023256	6.58E−13	0	0
	p17	−0.00484	−0.03787	−0.063965	−0.02945	0.024237	−0.03337		0.013551	−0.05265	−0.01987	0.12184	−0.0013			−0.00188	−0.02027	−0.00952	0.01679				
	p18			0.0042543	0.06849	0.15378	0.039219				−0.00846	0.042259	−0.01509		0.035242	0.03429	0.18159	0.091095	0.10839	0.028994			0.046521
	p19	0	0	−0.019928	0.019905	0.066681	0.053835	0.032972	−0.02321	0.013074	0.02538	0.002022	0.024326	0.025256	0.046121	0.000998	0.029176		−0.00074		0	0	0
	p20	0		0	−0.01654	−0.00233	−0.02132	−0.033931	0.063515	−0.06312	0.048455	−0.00645	−0.10108	0.016345	0.018334	−0.03267	0.087404	0.068676	0.033321	0	0	0	0

Verbal Before

Participants		Channels																					
Group	#	ch1	ch2	ch3	ch4	ch5	ch6	ch7	ch8	ch9	ch10	ch11	ch12	ch13	ch14	ch15	ch16	ch17	ch18	ch19	ch20	ch21	ch22
	p1	0.013106	0.054474	0.042237	0.07639	0.046835	0.000175	0.082822	−0.01705	0.034596		−0.05421	0.010274	−0.05083	0.021517	0.020635		0.048718	0.023636	0.085048	0	0	0
	p2	0.13818	0.065714	0.028681	−0.01041	0.049017	−0.0757			−0.08756			−0.01666	0.007148	−0.01615	−0.03191	−0.07536		−0.00384	−0.020175	0	0	0
	p3			0.077894	0.001006	0.10083	0.011872	0.19759	0.063238	−0.07626	−0.00419		−0.02071			0.002962			0.001818		0	−0.036113	0
	p4				0.007226	0.080632	0.12057			0.086195			0.050115	0.004326		0.075423						0	0
G_{alone}	p5	−0.04768	0.047013	−0.042802	−0.00895	−0.01464	−0.07337			−0.08627	−0.00461		0.048298	−0.09428		−0.00144		0.028009		0.0044663		−0.020057	
	p6	−0.04683	0.074474	0.038136	−0.00694	−0.04654	0.025837	0.07232	0.051711	−0.02109	0.012378	0.005751	0.015056	0.022735	0.031951	−0.00581	0.01488	0.02161	−0.02224	−0.003899	0	0	0
	p7			−0.12668	0.070584		0.02992			−0.02678	0.027462		0.083052			0.088289			0.010166				
	p8	0.013485	−0.02537	0.014674	0.042867	0.039515	0.015264	0.045126	−0.00454	0.14962	0.070918	−0.05075	0.098664	−0.06421	0.039523	0.01528	0.045676	−0.02503	0.018479	−2.60E−05			
	p9	0		−1.91E−08	−0.01463	0.065649	0.019592	0.13709	0.088504	0.034711	0.087415	0.088716	0.098333	0.047216	0.14869	0.052143	0.13028		0.10809			0	0
	p10	0.066293		0.0010321			0.019935		0.016151	0.006531	−0.02325	0.009472	−0.01341	0.036171	0.026916	0.061569	0.022468	0.037531	0.008826	0.0030717	0		0
	p11	0.052869		0.079654	0.058137			0		0	0.035824						−0.08834						
	p12	−0.03636	−0.00128	−0.032415	0.047537	−0.02809	0.03325	−0.079137	0.019272	0.001162	0.010566	0.012956	−0.04418	0.084559	0.10313	−0.04237	−0.02802	−0.00266	−0.02732	−0.029829	0	0	0
	p13	−0.05647	−0.0045	−0.027072	0.014696	0.05271	−0.04745	0.12355		−0.01795	−0.02107		−0.04788	0.053657		−0.02076			0.015216	0.023275		0.019809	0.013594
	p14			−0.030463	0.01676	−0.02355	0.13752	−0.019254	−0.01811	0.013003	−0.00067	−0.03014	−0.01155	−0.02153	0.022504	−0.01444	0.039152	−0.04591	−0.00623	0.015943	0	0	0
G_{f2f}	p15		−0.03119	0.057628	−0.04802	−0.0115	−0.03321		−0.01815	−0.03924	−0.04829	−0.07494	−0.0459	−0.08749	0.007286	−0.0428							
	p16	0		5.62E−16			0.011553	0.050537	−0.00428	−0.00692	−0.06682	0.017819	−0.05522	0.10832	−0.01213	−0.02466	0.035104	0.03457	−0.03919		−7.86E−57	0	0
	p17	0.053026	0.036422	0.021516	0.005402	0.009615	−0.0268		0.003539	−0.037	−0.05807		−0.02743			−0.08897	−0.06877	0.019126					
	p18			−0.01538	0.011829	0.080221	0.027762		0.05252	−0.07125	0.07226	0.07523	0.097566	0.067501	0.081177	0.075979	0.13464	0.14344	0.090465	0.071599			
	p19	0	0	0.0098318	0.015105	0.012346	0.075437	0.084664	0.018623	0.036436	0.014158	−0.01468	0.020478	0.03055	0.085739	0.041773	0.027447	0.022206	−0.00235	0.015992	0	0	0
	p20	0		0	−0.01583	−0.01489	−0.01139	0.012117	0.000658	0.008855		0.082692	0.062408	−0.01764	0.054362	0.018344	0.10842	0.068931		−0.022218	0	0	0

Verbal After

Participants		Channels																					
Group	#	ch1	ch2	ch3	ch4	ch5	ch6	ch7	ch8	ch9	ch10	ch11	ch12	ch13	ch14	ch15	ch16	ch17	ch18	ch19	ch20	ch21	ch22
	p1	−0.01429	−0.00788	0.005749	0.015204	−0.01005	−0.02152	−0.023033	0.099918	0.014982	−0.08874	0.010819	0.014803			0.004028	−0.03819	0.15296	0.19088	−0.025676	0	0	0
	p2	0.004656	−0.01171	−0.0046334	−0.03727	0.049008	0.0247			0.149			0.047123	−0.01236	−0.00815	0.10821	0.060441	−0.04303	0.1262	−0.13599	0	0	0
	p3	−0.0202		−0.038187		0.036953	−0.04127	0.0086326	−0.01166	−0.0129			−0.01301			0.031444			0.018507	0.14937	0	−1.46E−06	0
	p4				0.012838	0.017175	0.019716	0.043267	−0.04031	0.046266			−0.03039	0.094515		0.046812						0	0
G_{alone}	p5	−0.02288			0.10394	0.13823		−0.0012468		−0.00148			0.093112			0.066666		0.017353	0.089457	0.080563		0.029871	
	p6	0.046041	−0.02208	0.052693	−0.05222	0.007245	0.009635	−0.065141	0.053241	−0.0033	0.082649	0.00478	0.042452	0.02594	−0.03333	−0.03903	0.067401	0.005111	0.038399	0.036889	0	0	0
	p7				0.024285		0.099399	−2.44E−05		0.13419	−0.08578		−0.15067			0.061545			0.002273				
	p8	0.044444	−0.017	0.028569	0.000633	−0.0453	−0.00638	−0.14732	−0.00536	0.010913	−0.05038	0.11705	−0.05374	0.053177	0.034226	−0.05174	−0.04579	0.023255	0.055446	−2.40E−16		1.53E−260	5.94E−262
	p9	0		9.97E−101	0.026816	−0.08586	0.020549	0.089826	0.096822	0.10059	−0.07352	−0.10777	0.15205	0.012408	0.021512	0.018791	0.085782		0.052163		0	0	0
	p10			0.039791	0.10241		0.025396	−0.093835		0.069862	−0.0103	−0.03965	0.090932	−0.02999	−0.11501	0.024888	−0.10539	0.085931	−0.02054	−0.060233	0		0
	p11	0.045034	0.11077	0.037956	0.10859			0		0	−0.12812	0.014201			−0.00879								
	p12	−0.04137	−0.02425	−0.021895	0.041321	−0.0483	−0.01352	0.027138	0.022364	0.023579	−0.00172	0.11828	−0.00303	0.041787	0.049708	0.02612	0.017468	−0.00628	−0.00813	0.045675	0	0	0
	p13	−0.04754	0.012884	−0.013555	0.008888	−0.02472	0.057575	0.074578	−0.11556	−0.02235	−0.09348		−0.0614	−0.01653		−0.04979			−0.00632	−0.013094	0.040361	0.063471	0.019022
	p14			−0.012068	−0.00693	−0.04243	0.003368	0.014841	−0.01325	−0.04589	0.039747	−0.00032	−0.04703	−0.08867	0.07536	0.003985	−0.04317	0.000864	−0.00663	−0.009652	0	0	0
G_{f2f}	p15			0.044821	0.028575	0.047123	−0.05161	−4.59E−09	−0.05517	−0.04486	−0.06809	−0.10697	−0.05805	−0.07795	0.029544	−0.08665							
	p16	0		0			−0.02727	−0.094426	−0.12478	−0.09115	−0.0887	−0.10536	0.020008		−0.1462	−0.00788	−0.08072	0.006102	−0.02504	−0.11295	0.0002047	0	0
	p17	0.008925	0.089744	−0.052829	0.054862	0.08454	0.069785		0.045266	0.083004	0.018533	0.11338	0.065823			−0.03128	0.004681	0.062959	−0.03457				
	p18			0.071305	0.060145	−0.02354	−0.06802		−0.0898		−0.09603	−0.05717	−0.00394	0.017241	−0.03993	0.023776	−0.05399	−0.03581	0.006326	−0.008494			
	p19	0	0	0.0061308	0.066088	−0.00613	0.016516	0.013779	−0.00374	0.015429	−0.05832	0.005561	−0.0039	0.054685	0.073617	−0.00561	−0.00629	0.092156	−0.02766	0.12929	0	0	0
	p20	0		0	0.035499	0.045344	0.010339	0.03644	0.01932	0.04125	−0.04691	−0.06117	0.003519	−0.02518	−0.03664	0.008096	−0.04218	−0.02558	0.055636	0	0	0	0
