# Supplementary material for: Informal Face-to-Face Interaction Improves Mood State Reflected in Prefrontal Cortex Activity
Source: Front Hum Neurosci. 2016 May 3;10:194. doi: 10.3389/fnhum.2016.00194 (PMC4853847; doi:10.3389/fnhum.2016.00194)
Supplement: Data Sheet S3 — The mean accuracy of the five sequential WM tasks of verbal and spatial contents. [file DataSheet3.PDF]

Data Sheet S3

Task accuracy

WM scores

Participants		Before		After	
Group	#	Spatial	Verbal	Spatial	Verbal
G_{alone}	p1	60%	100%	100%	100%
	p2	100%	100%	100%	100%
	p3	100%	100%	80%	100%
	p4	100%	100%	100%	40%
	p5	100%	100%	100%	80%
	p6	80%	100%	100%	100%
	p7	100%	100%	100%	100%
	p8	100%	100%	100%	100%
	p9	100%	100%	100%	80%
G_{f2f}	p10	100%	100%	100%	100%
	p11	100%	100%	100%	100%
	p12	60%	80%	100%	100%
	p13				
	p14	60%	100%	80%	100%
	p15	100%	75%	80%	80%
	p16	80%	100%	100%	100%
	p17	100%	100%	100%	100%
	p18	100%	100%	100%	100%
	p19	100%	100%	100%	100%
	p20	100%	100%	80%	100%

Note: p3 was removed because of a failure to detect his button press event.
